# Assessing the impact of digital transformation on capital market information efficiency under environmental uncertainty: Evidence from China

**DOI:** 10.1371/journal.pone.0295187

**Published:** 2024-01-12

**Authors:** Tao Feng, Xiaohuan Dong, Yueyun Wang

**Affiliations:** 1 School of Economics and Management, Shanghai Maritime University, Shanghai, China; 2 School of Economics and Management, Ningxia University, Yinchuan City, Ningxia Province, China; 3 Xinhua College of Ningxia University, Yinchuan City, Ningxia Province, China; Yunnan Technology and Business University, CHINA

## Abstract

Digital transformation is an emerging development opportunity for enterprises in the digital economy and comprehensively reflects the application of digital technology to production, operation, and management strategies. This study used data on A-share listed enterprises in Shanghai and Shenzhen from 2007 to 2022 to examine the relationship between the digital transformation of enterprises and information efficiency of the capital market. Findings revealed that digital transformation can improve the information efficiency of the capital market and that environmental uncertainty plays a more significant regulatory role. The greater the environmental uncertainty, the more enterprises with a high degree of digital transformation can promote the information efficiency of the capital market. Additional analysis showed that the promotional effect of digital transformation on the information efficiency of the capital market is better for non-state-owned enterprises and small and medium-sized enterprises. This study provides detailed insights into digital transformation and capital market information efficiency, which enriches the research related to the economic consequences of digital transformation and demonstrates the theoretical and practical value of corporate digital transformation.

## 1 Introduction

The digital economy, as a new paradigm for guiding rapid and optimal resource allocation, has made a profound impact on China’s economy. It has fundamentally transformed public consumption patterns and lifestyle behaviors, becoming a driving force and engine for economic development while facilitating high-quality economic growth and resource regeneration. Currently China’s internet and digital economy are experiencing robust and rapid growth, leading to the generation of an enormous wealth of big data within the country. In 2019, the scale of China’s digital economy increased through digitalisation, reaching 35.8 trillion yuan, accounting for 36% of the gross domestic product. The digital scale is 28.8 trillion yuan, and the proportion of industry in the digital economy has risen from 49.1% in 2005 to 80.2%, which has become the main driving force for the development of a digital economy.

According to a white paper on the development of China’s digital economy, the scale of the country’s digital economy reached 39.2 trillion yuan in 2020, accounting for 38.6% of the gross domestic product, which has become an important support for the high-quality development of the national economy. As the micro-composition of the macro-economy, enterprises serve a crucial function in the development and transformation of the macro-digital economy, and digital transformation is gradually reflected in the specific production behaviour of enterprises. In addition to being a micro-transformation of digital technology and production development, the digital transformation of enterprises also represents the transformation of enterprises from traditional production systems to digital system-based operations.

The capital market is a market for trading and allocating capital resources that provides enterprises with channels and mechanisms for raising capital, structural adjustment, and optimisation. It serves as a reference for enterprise value evaluation and provides a convenient trading mechanism for price discovery and value realisation. The capital market is a significant part of the financial market, and financial markets, institutions, products and instruments, infrastructure, and market participants together constitute a huge and complex financial system. Accelerating the development of the capital market has profound practical significance and plays a unique role in the historical process of building a modern economic system in China. Capital market efficiency refers to the realisation of finance on the capital market, optimal allocation of resources, and a high degree of functionality. Achieving accurate and timely reflection of capital market information in share prices and enhancing the efficiency of capital market information are crucial requirements for achieving optimal resource allocation.

Against the backdrop of the vigorous development of the digital economy, the promotion of enterprise digital transformation has become an inevitable trend in the development of the market economy. It is foreseeable that the digital transformation of enterprises will have a far-reaching impact on micro enterprises and the macro economy (Maqsood U S,2023; Chanias S et al.,2019;Verhoef PC et al.,2021;Sousa-Zomer TT et al.,2020) [1–4]. An important function of the capital market is to guide the effective allocation of resources based on stock prices, and its functioning needs to be able to reflect the real information of enterprises (Huan L et al.,2023;Jun Huang and Zhaorui Guo, 2014; Bin G et al.,2023) [5–7]. An effective capital market can use price information to guide resource allocation and thus create value, therefore, the ability of this stock price to reflect information becomes an important means of measuring whether the capital market operates effectively (I. EA et al.,2022; Zhu Hongjun et al., 2007) [8, 9], and stock price synchronization is an important reflection of whether the price reflects the information of the company. Share price synchronization is essentially caused by information asymmetry between companies and investors, and digital transformation can play a unique role in improving information efficiency and monitoring. Therefore, exploring the relationship between the two can provide new ideas for improving the efficiency of capital markets, and it can also help to understand the impact of digital transformation in capital markets and provide inspiration for subsequent research. Digital transformation is more in line with the development trend of the digital economy era, and it is easier to attract the attention of analysts in the capital market, so that analysts can issue reports containing company-specific information, so that the transparency of enterprises can be effectively improved.The high level of investor concern will also make management more cautious when considering information manipulation, weakening their motivation and extent of information manipulation (Ran C et al.,2022; Wangyi C et al.,2021) [10, 11]. The high versatility and permeability of digital technology will lead to the realisation of zero distance between the enterprise and the stakeholder groups, and improve the efficiency of internal control simultaneously,improve the ability to interact between investors and management, thereby further enhancing information transparency and discouraging information manipulation, ultimately improving the efficiency of capital market information (Thomas B et al.,2021;Li Chao et al.,2022;Liao P et al.,2021) [12–14].

This study begins by exploring the relationship between digital transformation and capital market information efficiency. Additionally, it investigates how the relationship between these two factors changes in the context of environmental uncertainty. First, we use regression analysis to explore the impact of digital transformation on capital market information efficiency. Second, the moderating role of environmental uncertainty in digital transformation and capital market information efficiency is considered. Third, we examine the role of analyst attention and technological innovation inputs in digital transformation and capital market information efficiency. Fourth, through further analysis, the role of digital transformation on capital market information efficiency is examined using the nature of firm ownership and the grouping of firm sizes.

Existing literature exploring the relationship between capital markets and digital transformation mainly explores the relationship between digital transformation and the quality of accounting information, the risk of stock price crashes, etc., whereas this study explores the impact of digital transformation on the information efficiency of capital markets from the perspective of environmental uncertainty and discusses the mechanisms of impact. Therefore, the possible marginal contributions of this study are as follows: First, it analyses the relationship between“digital transformation and capital market information efficiency”after the introduction of environmental uncertainty variables. Second, it offers substantial reference and practical value to the theoretical study of the relationship between digital transformation and capital markets. Third, it provides incentives and motivation for firms of different sizes and types of property rights to implement digital transformation. Fourth, it provides complete theoretical support for the different roles of analysts’ attention and technological innovation inputs in the relationship between digital transformation and the capital market information environment.

In the baseline analysis, it was discovered that digital transformation suppresses stock price synchronisation and improves capital market information efficiency. Meanwhile, in the moderating effect test, it was found that the impact of digital transformation on capital market information efficiency is more significant when the external environment is at a higher level of uncertainty. In exploring the mechanisms of influence, the study reveals that analyst attention plays a significant mediating role in the relationship between digital transformation and capital market information efficiency. On the other hand, technological innovation input does not demonstrate a significant influence in this context. Further analysis, conducted by subdividing listed firms into large, small, and medium-sized firms, revealed that the impact of digital transformation on capital market information efficiency was more pronounced for small and medium-sized firms compared to large firms. In addition, the nature of firm ownership was also considered, and it was found that the digital transformation of non-state enterprises has a more significant effect on enhancing capital market information efficiency.

This paper comprises eight sections. The introduction provides an overview, followed by the literature review in the second section. The third section presents the research hypotheses, while the fourth section outlines the research design. The empirical analysis is presented in the fifth section, followed by the influence mechanism analysis in the sixth section. Additional analysis is included in the seventh section. The conclusion is summarized in the eighth section.

## 2 Literature review

### 2.1 Digital transformation

The existing literature on digital transformation is primarily addressed in terms of connotations, influencing factors, and consequences. From the connotation perspective, Rachinger et al. (2019) asserted that digitalisation is the application of digital technology, while digital transformation is defined as the process of restructuring the economy, institutions, and society at a systemic level [15]. Al Nuaimi Bader et al. (2022) highlighted that digital transformation is not simply the application of digital technology, nor is it simply investing in digital infrastructure to enhance profitability [16]. Loonam et al. (2018) found that, unlike the process of transformation or change in dynamic markets, digital technology accelerates the pace of change, leading to greater volatility, complexity, and uncertainty [17]. Liu et al. (2011) suggested that digital transformation is an organisational transformation that integrates digital technologies and business processes in the digital economy [18]. Tsai et al. (2022) argued that digital technologies, as part of transformation, are becoming a part of business strategy, organisational culture, and structure [19].

Currently, research on the relationship between digital transformation and the capital market has resulted in some substantial and enlightening findings, primarily at the macro and micro levels of the capital market. First, at the macro level of the capital market, Dai et al. (2022) explored the relationship between digital transformation and the opening of the capital market and assumed it was under the rising tide of the digital revolution [20]. Wessel et al. (2021) asserted that listed enterprises should promote the digital transformation of enterprises with the help of the two-way opening of the capital market system [21]. Second, starting with institutional investors in the capital market, Li (2021) explored their association with the digital transformation of enterprises and assumed that pressure-sensitive institutional investors inhibit the digital transformation of enterprises, while pressure-resistant institutional investors can effectively promote the digital transformation of enterprises [22]. Third, from the perspective of enterprises themselves, Xu et al. (2022) explored the association between financialization and digital transformation [23]. Gao et al. (2021) demonstrated that enterprise financialization will have a significant adverse effect on the financial behaviour, financing status, production input, and innovation output of enterprises, thus restraining digital transformation and analysing the relationship between digital transformation and the risk of stock price collapse in enterprises [24].

GQ Wang (2023) empirically investigated the relationship between digital transformation and trade credit provision and found that digital transformation significantly increases the supply of trade credit and that the mechanism for this relationship is an increase in short-term bank credit [25]. Niu et al. (2023) analysed the impact of digital transformation on corporate innovation and found that digital transformation positively impacts corporate innovation. Mechanistic tests show that digital transformation helps to alleviate corporate financial constraints and improve corporate governance, thereby breaking down barriers to corporate innovation [26]. Xu et al. (2023) empirically studied the impact of inefficient corporate investment on digital transformation and found that inefficient investment is detrimental to increased digitalisation. The greater the financing pressure on firms, the worse the digital transformation, and this negative effect is exacerbated by financing constraints [27].

### 2.2 Capital market information efficiency

As an important indicator of capital market information efficiency, stock price synchronisation, also known as “same up, same down” is described by Mork et al. (2000) as the relationship between a company’s stock price volatility and market and industry volatility, measuring the extent to which a company’s stock price contains information about its own characteristics [28]. Omar et al. (2016) argued that excessive stock price synchronisation can severely damage capital market resource allocation efficiency [29], while Song (2015) maintained that stock price synchronisation increases the risk of stock price collapse [30].

The existing literature on capital market information efficiency has been studied primarily from the perspective of influencing factors and economic consequences. From the perspective of influencing factors, numerous factors affect information efficiency in capital markets. Tian et al. (2022) argued that, as a complement or alternative, the media influences the pricing of securities and the cost of capital through the widespread dissemination of information [31]. Jia et al. (2020) suggested that local media can directly improve capital market information efficiency; however, compared to local media, central media can also indirectly improve capital market information efficiency by weakening the negative relationship between political affiliation and capital market information integration [32]. Xu et al. (2022) suggested that questioning regulation significantly reduces the share price synchronisation of questioned companies, making their share prices more reflective of firm-level idiosyncratic information and improving the information efficiency of the capital market [33]. Wu et al. (2022) found that analysts’ positive surplus forecast revisions significantly reduced the synchronisation of company share prices, and the effect was more pronounced among star, non-underwriter, and female analysts [34]. Ruan (2021) suggested that an extensible business reporting language, as a common standard for global business reporting, can compensate for the shortcomings of traditional financial reporting in terms of regulatory disclosure and information usage costs [35]. Nan et al. (2018) argued that the financing and financing transactions, contrary to the original policy intention, failed to achieve the desired objective of enhancing information efficiency in the capital market. Furthermore, the implementation of the financing and financing system not only failed to reduce stock price synchronization among relevant securities but also increased their synchronization [36].

It can be observed that most of the existing literature discusses the factors influencing the information efficiency of capital markets from the perspectives of self-media disclosure, inquiry regulation, analysts’surplus forecasts, extensible business reporting language, financing transactions, and so on, but not the association of digital transformation with the information efficiency of capital markets.

## 3 Research hypothesis

### 3.1 Digital transformation and capital market information efficiency

According to the efficient market theory, when the market fails to reach full efficiency, stock prices cannot reflect all the information at the enterprise level. In comparison to mature markets in the West, China’s market systems are still relatively imperfect, requiring improvements to enhance resource allocation efficiency. Consequently, China’s market is characterized by a greater abundance of noisy information and irrational behavior. In other words, stock price fluctuations in China may not necessarily be driven solely by the specific information content of individual enterprises. The stock market in China is characterized by prevalent phenomena such as chasing up and killing down, the herd effect, and other behavioral biases. These factors contribute to a significant presence of noise trading behavior, indicating that the market type leans towards being inefficient. Most participants in China’s stock market are small- and medium-sized investors who lack professional investment knowledge and information discrimination abilities and are very easily deceived by bankers. In other words, the stock price of enterprises on the capital market is not necessarily the actual performance of each enterprise; that is, the synchronisation of stock prices is low, which reduces the efficiency of the capital market, thus market effectiveness is weak. Therefore, the research hypothesis H1a is proposed.

H1a: The higher the degree of digital transformation implemented by enterprises, the more stock price synchronisation there will be, thus improving the information efficiency of the capital market.

However, considering the Solow paradox, or productivity paradox, which primarily came from the United States in the late 1980s, scholar Strassman surveyed 292 enterprises and found that IT Investment and Return on Investment (ROI) have no expected or obvious correlation. Robert Solow, winner of the 1987 Nobel Memorial Prize in Economic Sciences, referred to this phenomenon as the ‘productivity paradox’, or Solow’s paradox. The implication is that, despite investment in information and communication technology resources, the effectiveness of examining statistical productivity is minimal. Solow compared the growth of total factor productivity and labour productivity in the United States between 1948 and 1973 and 1973–1998 and found that information technology represented by computers did not initiate the improvement of total factor productivity in the United States but showed a downward trend. Additionally, following Solow’s paradox, a paradox in the digital transformation of enterprises may arise. Despite substantial investments in information technology, including funds, manpower, and other resources, enterprises focusing on digital transformation may not achieve the anticipated return on investment. This may result in a lack of exceptional financial performance and stock price improvement, distinguishing them from other enterprises. Consequently, the expected reduction in stock price synchronization and enhancement of information efficiency in the capital market may not materialize.

Furthermore, in line with the herd effect theory, the implementation of digital transformation by certain enterprises may lead to an increase in their stock prices. Subsequently, other enterprises and the market as whole may blindly follow suit. This phenomenon can disrupt and influence analysts’earnings forecasts and judgements, as well as investors’decision-making processes. While these occurrences may contribute to the synchronization of stock prices, they do not guarantee or enhance the overall effectiveness of the capital market.Based on this, the following research hypothesis is proposed:

H1b: A negative relationship exists between the degree of digital transformation implemented by enterprises and stock price synchronization, leading to a decrease in the information efficiency of the capital market.

### 3.2 Moderating effects of environmental uncertainty

Chen (2022) proposed that the development of digital transformation is sensitive to changes in the external environment, thus environmental uncertainty is added to the model as a moderating variable in this study [37]. Environmental uncertainty refers to the state in which enterprise managers cannot accurately predict changes in future technology, markets, and other environments when they do not fully understand external environmental information. Not all business activities of enterprises can be conducted alone, and they must rely on a certain environment and respond to its changes.

According to the theory of resource dependence, the scarce resources that enterprises rely on for survival are sourced from the environment, and uncertainty in the environment leads to a lack of control over key scarce resources. Therefore, when the degree of environmental uncertainty is high, enterprises need to establish a good relationship with the environment to obtain control of scarce resources.The information disclosed by enterprises serves as a crucial source of decision-making information for analysts when forecasting earnings. Consequently, in times of environmental uncertainty, other enterprises engage in more discussions concerning adjustments to strategies, positioning, and products [38]. At this time, enterprises with a higher degree of digital transformation will better reflect the advantages of moving to the technological frontier, create more corporate value, and raise stock prices to distinguish them from other enterprises that have not implemented or are less involved in digital transformation. Based on the above, the research hypothesis H2a was proposed:

H2a: In the presence of greater environmental uncertainty, enterprises with a high degree of digital transformation can restrain the synchronisation of stock prices and improve the information efficiency of the capital market.

Therefore, March (1991) believed that based on the uncertainty of the future business environment, the company would seek a relatively satisfactory solution rather than an optimal solution [39], that is, it would choose a conservative strategy rather than a radical strategy to control risks, thus it is possible to choose to reduce the work of digital transformation when the environmental uncertainty is relatively strong, thus reducing the degree of digital transformation, thereby improving the synchronisation of stock prices, and reducing the information efficiency of the capital market. Based on the above, the research hypothesis H2b was proposed:

H2b: In the presence of greater environmental uncertainty, enterprises with a low degree of digital transformation will improve the synchronisation of stock prices and reduce the information efficiency of the capital market.

## 4 Research design

### 4.1 Sample selection and data processing

Considering that China implemented the new accounting standards for business enterprises in 2007, this study selects the data of A-share listed enterprises in Shanghai and Shenzhen from 2007 to 2022 as the initial research sample, and in the acquisition of data for digital transformation, enterprises are classified into high-tech enterprises and non-high-tech enterprises, and then high-tech enterprises are further classified into specific categories, and the following treatment is carried out in the processing of the data: Firstly, financial enterprises are removed. Firstly, financial enterprises were excluded. Firstly, financial enterprises were excluded. Secondly, enterprises classified as ST or *ST and enterprises delisted during this period were excluded. Third, enterprises with a gearing ratio greater than 1 are excluded. Fourth, only samples with no missing data for at least five consecutive years are retained in this study. Fifth, to reduce the effect of outliers, this study reduces the tails of all micro-level continuous variables by 1% and 99%. The raw data are obtained from the China Securities Market and Accounting Research (CSMAR) database, the annual reports of the examined firms are obtained from the official websites of the Shenzhen Stock Exchange and the Shanghai Stock Exchange, and other data are obtained using Python software. The process of data selection and processing is shown in Table 1.

**Table 1:** Sample selection:Obtaining data samples categorised by industry type.

Constructions of stock price information content data for firm-year	Numbers
Observations with data in CSMAR for 2007–2022	40573
• Listed companies in financial industry	(1130)
• Special treatment listed companies	(1204)
• Leverage not below 1	(1399)
• Data listed less than five years	(798)
Sample with stock price information content data	36042
Constructions of digital transformation data for firm-year:Electronic Information Technology	Numbers
Observations with data in CSMAR for 2007–2022	4564
• Data on control variables missing	(550)
• Special treatment listed companies	(605)
• Leverage not below 1	(489)
• Data listed less than five years	(309)
Sample with digital transformation data	2611
Constructions of digital transformation data for firm-year:Biological and New Pharmaceutical Technologies	Numbers
Observations with data in CSMAR for 2007–2022	3890
• Data on control variables missing	(540)
• Special treatment listed companies	(402)
• Leverage not below 1	(380)
• Data listed less than five years	(400)
Sample with digital transformation data	2168
Constructions of digital transformation data for firm-year:Aerospace Technology	Numbers
Observations with data in CSMAR for 2007–2022	3140
• Data on control variables missing	(590)
• Special treatment listed companies	(408)
• Leverage not below 1	(616)
• Data listed less than five years	(400)
Sample with digital transformation data	1126
Constructions of digital transformation data for firm-year:New Material Technology	Numbers
Observations with data in CSMAR for 2007–2022	3771
• Data on control variables missing	(452)
• Special treatment listed companies	(204)
• Leverage not below 1	(399)
• Data listed less than five years	(410)
Sample with digital transformation data	2306
Constructions of digital transformation data for firm-year:High-tech Service Industry	Numbers
Observations with data in CSMAR for 2007–2022	3904
• Data on control variables missing	(289)
• Special treatment listed companies	(215)
• Leverage not below 1	(377)
• Data listed less than five years	(287)
Sample with digital transformation data	2736
Constructions of digital transformation data for firm-year:New energy and energy-saving technologies	Numbers
Observations with data in CSMAR for 2007–2022	3557
• Data on control variables missing	(275)
• Special treatment listed companies	(344)
• Leverage not below 1	(199)
• Data listed less than five years	(252)
Sample with digital transformation data	2487
Constructions of digital transformation data for firm-year:Resource and Environmental Technology	Numbers
Observations with data in CSMAR for 2007–2022	2304
• Data on control variables missing	(350)
• Special treatment listed companies	(219)
• Leverage not below 1	(305)
• Data listed less than five years	(200)
Sample with digital transformation data	1230
Constructions of digital transformation data for firm-year:High-tech transformation of traditional industries	Numbers
Observations with data in CSMAR for 2007–2022	3087
• Data on control variables missing	(542)
• Special treatment listed companies	(490)
• Leverage not below 1	(471)
• Data listed less than five years	(358)
Sample with digital transformation data	1226
Constructions of digital transformation data for firm-year:Non-high-tech enterprises	Numbers
Observations with data in CSMAR for 2007–2022	7729
• Data on control variables missing	(1130)
• Special treatment listed companies	(877)
• Leverage not below 1	(903)
• Data listed less than five years	(700)
Sample with digital transformation data	4119

This table presents the procedure of the sample selection among the main variables in the research.

### 4.2 Variable setting

1. Explained variable
  Following Kong (2015) and Yang (2018), this study used stock price synchronisation to represent capital market information efficiency [40, 41]. The lower the stock price synchronisation, the better the capital market information efficiency. Drawing on Durnev et al. (2004), this study used the following model to estimate the R^2^ of individual stocks [42].
$$R_{i,t}=\alpha_{i}+\beta_{i.1}R_{m},\;t+\beta_{i.2}R_{i,t}+\varepsilon_{i.t}$$ (1)
  where R_i,t_ is the yield of stock i in week t, R_m,t_ is the yield of the market index in week t, and R_i,t_ is the yield of industry i in week t, weighted by the circulation market value of enterprises in the industry with reference to the industry classification standard of the China Securities Regulatory Commission. Then, the goodness of fit R^2^ generated by the regression of model (1) has a normal distribution, and the logarithmization of Formula (2) is used to obtain the stock price synchronisation index.
$${\mathrm{SYNCH}}_{i,t}=\ln\;(R^{2}{}_{i,t}/(1‐R^{2}{}_{i,t})$$ (2)
2. Explanatory variable
  Jiang (2022) used a 0–1 dummy variable of "whether the enterprise is undergoing digital transformation" to measure the digital transformation of enterprises [43]. However, this technical treatment does not effectively show the intensity of enterprise digital transformation and thus can easily lead to a misjudgement of the degree of transformation. This study argues that corporate digital transformation, as a major strategy for high-quality corporate development in the new era, is greatly reflected in the annual reports and announcements of enterprises due to its high generality and guiding nature. Therefore, this study uses the frequency of occurrence of key words related to digital transformation in the annual report of the i company on CSMAR.The list of keywords for digital transformation is presented in S1 Table.
3. Moderating variables
  Environmental uncertainty (EU). First, each enterprise’s data for the past five years were run through Eq (3) using the ordinary least squares method to estimate the abnormal sales proceeds of the past five years. Second, the standard deviation of abnormal sales proceeds in the past five years divided by the average sales proceeds in the past five years was used as the raw measure of environmental uncertainty, as shown in Eq (4).
$$Sale=\varphi_{0}+\varphi_{1}Year+\varepsilon$$ (3)
$${EU(\varepsilon_{i,t})}_{raw}=\frac{\sqrt{\sum_{t=1}^{5}\frac{{\left(\varepsilon_{i,t}-\bar{\varepsilon }\right)}^{2}}{5}}}{\bar{Sale}}$$ (4)
  *Sale* is the sales proceeds, *Year* is the annual variable, *ε* is the abnormal sales proceeds. *EU(ε_i,t_)*_*raw*_, the unadjusted enterprise-specific measure of environmental uncertainty, is the coefficient of variation for abnormal sales proceeds calculated over a five-year period. *Sale* is the average sales proceeds of enterprise *i* in the past five years, *ε_i,t_* is the abnormal sales proceeds of enterprise *i* in year *t*, and $\bar{\varepsilon }$ is the average abnormal sales proceeds of enterprise *i* in the past five years. Industry environmental uncertainty was then measured as the unadjusted median of environmental uncertainty for all enterprises in the same industry in the same year. Finally, to mitigate the industry effect, environmental uncertainty (*EU*_*i*,*t*_) was defined as the unadjusted measure of environmental uncertainty (*EU(ε_i,t_)*_*raw*_) divided by the industry environmental uncertainty.
4. Control variables
  This study used the following control variables: enterprise size (SIZE), age of the listed enterprise (Age), book_to_market (BM), audit opinion type (AUDIT), gross operating income growth rate (growth), leverage ratio (Lev), shareholding ratio of the largest shareholder (Top1), equity concentration (Shrcr), annual stock turnover rate (VOL), net profit margin on total assets (ROA), return on net assets (ROE), institutional investor shareholding ratio (InsInvestor), board size (Board), whether auditors were from Big Four accounting firms (Big4), and Dual (indicating whether the general manager also served as the chairman, with ‘yes’ coded as 1, and ‘no’ coded as 0). Table 2 provides an explanation for each variable.

**Table 2:** Variable definition.

Variable Names	Definition
SYNCH	Individual stocks, comprehensive markets and industries adopt the return rate of reinvestment considering cash dividends, and the market and industry adopt the weighted average method of current market value to calculate the information content of share prices.
SYNCH_2	Individual stocks, comprehensive markets and industries all adopt the return rate considering the reinvestment of cash dividends, and the market and industry adopt the weighted average method of total market value to calculate the information content of share prices.
EDT	Use the frequency of occurrence of key words related to digital transformation in the annual report of the i company on CSMAR.
L2.EDT	The degree of digital transformation of enterprises lagging behind phase II。
L3.EDT	The degree of digital transformation of enterprises lagging behind three phases。
L4.EDT	The degree of digital transformation of enterprises lagging behind four phases.
Follow	Analyst attention +1, taking natural logarithm
Innov	The ratio of R & D investment to operating income of the I enterprise in year t
EU	Adjusted indicators of environmental uncertainty
VOL	The stock turnover rate of enterprise I in year t is calculated based on the number of circulating shares
InsInvestor	Share ownership percentage of institutional investors.
Lev	Leverage computed as total liabilities divided by total assets.
Roa	The net profit divided by average total assets.
Size	The natural logarithm of total assets of the firm at the end of the year.
Book_to_Market	Book value per share and market value per share.
Dual	The natural logarithm of the number of analysts covered the firm i in year t.
Growth	The growth rate of revenue of firm i in the previous year.
Age	The age of the listed company i.
Soe	Whether or not a state-owned enterprise, if so, state = 1, otherwise = 0.
Big4	Equal to 1 if firm i audited by one of the international Big 4 audit firms and 0 otherwise.
Shrcr	The shareholding concentration of enterprise I in year t is the shareholding ratio of the top ten shareholders.
Top1	Shareholding ratio of the largest shareholder of enterprise I in year t
Board	The size (number) of the board of directors of enterprise I in year t is logarithmicized.
Audit	In the audit opinion of enterprise I in year t, the opinion type is standard unqualified opinion, which is taken as 1, and others are taken as 0.
Industry	A dummy variable, if year t belongs to the year, it will be 1, otherwise it will be 0.
Year	A dummy variable, if company i belongs to the industry, it will be 1, otherwise it will be 0.

### 4.3 Research model

According to the above analysis, to empirically evaluate the relationship between the digital transformation of A-share listed enterprises in Shanghai and Shenzhen, the information efficiency of the capital market, and the regulatory role of environmental uncertainty, this study constructed a multilevel regression model in five steps:

Step 1 was to analyse the relationships between stock price synchronisation and the control variables and establish the basic model of the study.


$${\mathrm{SYNCH}}_{\mathrm{it}}=\lambda_{0}+\lambda_{1}{\mathrm{Size}}_{\mathrm{it}}+\lambda_{2}{\mathrm{Age}}_{\mathrm{it}}+\lambda_{3}{\mathrm{BM}}_{\mathrm{it}}+\lambda_{4}{\mathrm{Growth}}_{\mathrm{it}}+\lambda_{5}{\mathrm{Lev}}_{\mathrm{it}}+\lambda_{6}{\mathrm{Shrcr}}_{\mathrm{it}}+\lambda_{7}{\mathrm{VOL}}_{it}+\lambda_{8}{\mathrm{ROA}}_{\mathrm{it}}+\lambda_{9}{\mathrm{Inshold}}_{it}+\lambda_{10}{\mathrm{Board}}_{\mathrm{it}}+\lambda_{11}{\mathrm{Dual}}_{it}+\lambda_{12}{\mathrm{Big}}_{it}+\varepsilon_{it}$$ (5)

In Formula (3), i represents the enterprise, t represents the year, and ε_it_ represents the error term.

Step 2 was to verify the relationship among enterprise digital transformation, the synchronisation of stock prices, and the efficiency of the capital market. The regression model is shown in Formula (6). If the coefficient a_1_ of digital transformation is significantly negative, H1A is supported, and digital transformation can significantly inhibit the synchronisation of stock prices and improve the information efficiency of the capital market.


$${\mathrm{SYNCH}}_{\mathrm{it}}=a_{0}+a_{1}{\mathrm{EDT}}_{\mathrm{it}}+a_{2}{\mathrm{Size}}_{\mathrm{it}}+a_{3}{\mathrm{Age}}_{\mathrm{it}}+a_{4}{\mathrm{BM}}_{\mathrm{it}}+a_{5}{\mathrm{Growth}}_{\mathrm{it}}+a_{6}{\mathrm{Lev}}_{\mathrm{it}}+a_{7}{\mathrm{Shrcr}}_{\mathrm{it}}+a_{8}{\mathrm{VOL}}_{\mathrm{it}}+a_{9}{\mathrm{ROA}}_{\mathrm{it}}+a_{10}{\mathrm{Inshold}}_{\mathrm{it}}+a_{11}{\mathrm{Board}}_{\mathrm{it}}+a_{12}{\mathrm{Dual}}_{\mathrm{it}}+a_{13}\mathrm{Big}4_{\mathrm{it}}+\varepsilon_{\mathrm{it}}$$ (6)

Step 3 was to analyse the relationship between environmental uncertainty, digital transformation, and the information efficiency of the capital market. Environmental uncertainty and the interaction between digital transformation and environmental uncertainty were introduced on the basis of Formula (6). The regression model is shown in Formula (7). If the coefficient c_3_ of the interaction term between digital transformation and environmental uncertainty is significantly negative, hypothesis 2a is supported, and environmental uncertainty enhances the inhibitory effect of digital transformation on stock price synchronisation and improves the information efficiency of the capital market.


$${\mathrm{SYNCH}}_{\mathrm{it}}=c_{0}+c_{1}{\mathrm{EDT}}_{\mathrm{it}}+c_{2}{\mathrm{EU}}_{\mathrm{it}}+c_{3}\mathrm{EDT}\_{\mathrm{eu}}_{\mathrm{it}}+c_{4}{\mathrm{Size}}_{\mathrm{it}}+c_{5}{\mathrm{Age}}_{\mathrm{it}}+c_{6}{\mathrm{BM}}_{\mathrm{it}}+c_{7}{\mathrm{Growth}}_{\mathrm{it}}+c_{8}{\mathrm{Lev}}_{\mathrm{it}}+c_{9}{\mathrm{Shrcr}}_{it}+c_{10}{\mathrm{VOL}}_{it}+c_{11}{\mathrm{ROA}}_{it}+c_{12}{\mathrm{Inshold}}_{it}+c_{13}{\mathrm{Board}}_{it}+c_{14}{\mathrm{Dual}}_{it}+c_{15}\mathrm{Big}4_{it}+\varepsilon_{it}$$ (7)

In addition, fixed effects are used in the empirical design.

## 5 Empirical analyses

### 5.1 Model diagnosis

The variance inflation factors (VIFs) of the variables in the model were calculated to assess for possible multicollinearity. The results showed that none of the variance inflation factors (VIFs) exceeded 2 with a mean value below 1.45, indicating that there was no serious multicollinearity in the model. The White’s test was used to test the presence of heteroskedasticity, yielding a probability of prob>chi2 of 0.0632, which is greater than 0.05. It indicates that there is no heteroskedasticity. The test was used to examine the possible autocorrelation, yielding a probability of 0.0711 for chi2, which is greater than 0.05. It suggests that there is no autocorrelation in the research model.

### 5.2 Descriptive statistics

Panel A and B of Table 3 presents the descriptive statistics for 4436 enterprises and 20,009 non-balanced enterprise-year observations. Table 3 reports the mean, standard deviation, minimum, and maximum of all the main variables in this study. Variables marked with * are scaled by 100 to facilitate the presentation of descriptive statistics. The mean and median for each return metric are all near equality, suggesting fair, symmetrical return distributions. In panel A, the statistical results of EDT show that the average value of the degree of digital transformation of each enterprise is about 1.407, with the maximum value of 3.033 and the minimum value of 0. The gap is large, which also indicates that there are still enterprises that have not implemented digital transformation in state-owned enterprises, and that the level of digital transformation of state-owned enterprises is uneven, with the median value of 1.055, which indicates that enterprises that have carried out digital transformation in state-owned enterprises The overall level is low, and there are more enterprises in the middle and lower reaches of the level.B In panel B, the statistical results of EDT show that the degree of digital transformation of each enterprise is about 1.845, of which the maximum value is 5.182, and the minimum value is 2.055, which is a relatively small gap, indicating that the level of digital transformation of non-state-owned enterprises is high, and the median value is 2.972, indicating that the overall level is high, and most of the enterprises are in the upper-middle level. Combining the descriptive statistics of the two panels to analyse the data, it is found that the standard deviation of the asset size of the enterprises is large, with a minimum value of 18.551 and a maximum value of 27.149, indicating that the asset sizes are more different. Moreover, there is a gap between the performance of the environmental uncertainty variable in the two panels, indicating that the performance of SOEs and non-SOEs in the face of environmental uncertainty is not the same, so it is worthwhile to speculate whether the digital transformation work can still be carried out successfully under the effect of environmental uncertainty, as well as the impact on the capital market. In addition, after the variance expansion factor test of each variable, the VIF value of each variable was much lower than 10, indicating the absence of multicollinearity problem between the variables.

**Table 3:** Descriptive statistics.

**Panel A (State-owned enterprises)**												
Variable	N			Mean		P50		Standard deviation		Max		Min
SYNCH	12443			0.584		0.501		0.390		1.034		0.040
EDT	12443			1.407		1.055		0.169		3.033		0.000
EU	12443			0.267		0.187		0.191		0.963		0.020
VOL	12443			5.689		5.107		5.224		24.055		0.488
InsInvestor	12443			0.412		0.498		0.290		0.972		0.004
Lev	12443			0.417		0.493		0.267		0.983		0.078
Roa	12443			0.033		0.039		0.075		0.328		-0.224
Size	12443			23.006		21.188		1.925		26.092		18.551
Book_to_Market	12443			0.703		0.699		0.280		1.179		0.154
Dual	12443			0.277		0.000		0.418		1.000		0.000
Growth	12443			0.189		0.120		0.454		3.280		-0.602
Age	12443			2.096		2.188		0.807		3.297		0.000
Big4	12443			0.090		0.000		0.340		1.000		0.000
Shrcr	12443			0.419		0.585		0.175		0.890		0.212
Top1	12443			0.340		0.304		0.131		0.709		0.073
Board	12443			2.169		2.188		0.210		2.733		1.600
Audit	12443			0.954		1.000		0.219		1.000		0.000
**Panel B (Non-state-owned enterprises)**												
Variable		N	Mean		P50		Standard deviation		Max		Min	
SYNCH		7566	0.414		0.411		0.213		0.877		0.019	
EDT		7566	2.845		2.972		1.377		5.182		2.055	
EU		7566	0.167		0.117		0.161		0.965		0.014	
VOL		7566	6.708		5.158		5.316		27.028		0.470	
InsInvestor		7566	0.438		0.454		0.248		0.917		0.003	
Lev		7566	0.449		0.439		0.216		0.987		0.055	
Roa		7566	0.037		0.038		0.074		0.228		-0.305	
Size		7566	22.419		22.178		1.496		27.149		19.373	
Book_to_Market		7566	0.612		0.606		0.250		1.159		0.104	
Dual		7566	0.279		0.000		0.449		1.000		0.000	
Growth		7566	0.188		0.121		0.458		3.261		-0.643	
Age		7566	2.075		2.197		0.903		3.296		0.000	
Big4		7566	0.073		0.000		0.260		1.000		0.000	
Shrcr		7566	0.586		0.596		0.154		0.910		0.222	
Top1		7566	0.342		0.319		0.149		0.745		0.084	
Board		7566	2.139		2.197		0.211		2.708		1.609	
Audit		7566	0.955		1.000		0.207		1.000		0.000	

Note: This table exhibits the descriptive statistics for the variables used in regression model. All variables are as defined in Table 2.

### 5.3 Correlation analysis

Table 4 reports the correlation coefficients of the main variables in this study. The statistical results show a significant negative correlation between the information efficiency of the capital market (stock price synchronisation) and the degree of digital transformation (r = -0.149, P < 0. 01), and a significant negative correlation with the annual stock turnover rate (r = -0.139, P < 0. 01). There is a significant positive correlation between the degree of digital transformation and the scale of enterprises (r = 0.011, P < 0. 01), and a significant negative correlation with the asset liability ratio (r = -0.029, P < 0. 01). There is a significant positive correlation between the degree of digital transformation and ownership concentration (r = 0.098, P < 0. 01). It shows that the scale and ownership concentration of enterprises are positively promoting the digital transformation of enterprises to a certain extent, while the debt ratio is inversely hindering the degree of digital transformation.

**Table 4:** Correlation matrix.

	SYNCH	EDT	EU	InsInvestor	VOL	Lev	Size	Book_to_Market	Growth
SYNCH	1.000								
EDT	-0.030***	1.000							
EU	0.003*	-0.004*	1.000						
InsInvestor	0.111***	-0.131***	0.029***	1.000					
VOL	-0.129***	0.097***	-0.028***	-0.294***	1.000				
Lev	0.035***	-0.077***	0.010***	0.212***	-0.131***	1.000			
Size	0.243***	0.020***	0.014***	0.332***	-0.218***	0.482***	1.000		
Book_to_Market	0.102***	-0.102	0.018***	0.141***	-0.014	0.265	0.388***	1.000	
Growth	0.037***	-0.050***	0.002	0.296***	0.172***	0.272***	0.209***	-0.003**	1.000
Board	-0.139***	0.088***	-0.028***	-0.290***	-0.139***	-0.230***	-0.144***	0.010***	-0.169***
Age	0.097***	-0.067***	0.058***	0.237***	0.314***	0.343***	0.143***	-0.018***	0.152***
Top1	0.020***	-0.034***	0.006***	0.074***	0.051***	0.066***	0.047***	0.002***	0.049***
Shrcr	-0.016**	0.024***	-0.030***	-0.032***	-0.054***	-0.032***	-0.017**	0.005***	-0.020***
Roa	0.023***	-0.001	0.011	0.107***	-0.192***	-0.003	-0.161***	0.251	0.028***
Dual	-0.082***	0.119***	-0.012***	-0.225***	-0.135***	-0.163***	-0.128***	0.017**	-0.205***
Big4	0.128***	-0.020***	0.020***	0.283***	0.168***	0.339***	0.199***	-0.008	0.198***
Audit	0.056***	-0.011	-0.002	0.068***	-0.096***	0.036***	0.049***	0.104***	0.037***
	Board		Age	Top1	Shrcr	Roa	Dual	Big4	Audit
SYNCH									
EDT									
EU									
InsInvestor									
VOL									
Lev									
Size									
Book_to_Market									
Growth									
Board	1.000								
Age	-0.281***		1.000						
Top1	-0.020***		-0.028***	1.000					
Shrcr	0.068***		-0.254***	0.620***	1.000				
Roa	-0.007		-0.125***	-0.009	0.025***	1.000			
Dual	0.126***		-0.226***	-0.025***	0.054***	0.026***	1.000		
Big4	-0.120***		0.039***	0.059***	0.038***	0.024***	-0.081***	1.000	
Audit	-0.003		-0.084***	0.012*	0.038***	0.361***	0.002	0.052***	1.000

### 5.4 Baseline analysis

Baseline analysis of Table 5 demonstrates the results of the baseline regression, and in order to test the accuracy of the baseline model, this paper adopts the strategy of progressive regression. In M1, only time and industry fixed effects are controlled, and no control variables are added, and the results show that the regression coefficient of EDT is significantly negative (t = -2.71,p<0.01), indicating that digital transformation has a positive driving effect on the information efficiency of the capital market, and has a significant inhibitory effect on the synchronicity of stock prices, which preliminarily verifies the H1a. further, adding control variables in column (2), the EDT coefficient is reduced, considering that it is due to the fact that some of the factors affecting the information efficiency of the capital market are absorbed because of the presence of the control variables. However, the coefficient of EDT is still significantly negative (t = -2.63,p<0.01). To ensure the robustness of the findings, the squared term of the core explanatory variable digital transformation is added in column (3) for the non-linear relationship test, which shows that there is no non-linear relationship between EDT and SYNCH. Overall, as the level of digital transformation increases, stock price synchronisation decreases significantly and capital market information efficiency increases significantly.

**Table 5:** Test of H1: Baseline analysis.

		Linear relationship test					Non-linear relationship test				
		(1)		(2)			(3)				
Variables		SYNCH		SYNCH			SYNCH				
EDT		-0.0319***		-0.0412***			-0.0429***				
		(-2.71)		(-2.63)			(-2.88)				
EDT^2^							-0.1997				
							(-1.02)				
InsInvestor				-0.0311***			-0.0300***				
				(-5.17)			(-3.98)				
Lev				-0.1266***			-0.1285***				
				(-19.48)			(-15.19)				
Size				0.3491***			0.3373**				
				37.03			(28.60)				
Book_to_Market				0.0460***			0.0460***				
				(7.51)			(7.51)				
Growth				-0.0326***			-0.0326***				
				(-7.77)			(-7.77)				
Board				0.0081			-0.0004				
				(1.43)			(-0.06)				
VOL				-0.0142***			-0.0155**				
				(-2.92)			(-2.53)				
Age				0.0555***			0.0575***				
				(8.78)			(7.34)				
Top1				-0.0036			-0.0106				
				(-0.54)			(-1.24)				
Shrcr				0.0201***			0.0267***				
				(3.00)			(3.07)				
Roa				0.0152***			0.0127*				
				(2.81)			(1.87)				
Dual				-0.0040			-0.0089				
				(-0.35)			(-0.61)				
Big4				-0.0233			-0.0069				
				(-0.98)			(-0.23)				
Audit				0.1089***			0.1116***				
				(4.77)			(3.70)				
Industry		Yes		Yes			Yes				
Year		Yes		Yes			Yes				
Constant		0.7243***		0.8431***			0.7821***				
		(7.74)		(5.49)			(6.89)				
Observations		20009		20009			20009				
R-sq		0.4460		0.5060			0.4543				
Adjusted R-sq		0.4442		0.4902			0.4513				
**Panel A**											
Model		(1)		(2)			(3)				
Variables		SYNCH		SYNCH			SYNCH				
EDT				-0.0281***			-0.0225***				
				(-2.97)			(-2.78)				
InsInvestor		-0.0311***					-0.0300***				
		(-5.17)					(-3.98)				
Lev		-0.1266***					-0.1285***				
		(-19.48)					(-15.19)				
Size		0.3491***					0.3373**				
		37.03					(28.60)				
Book_to_Market		0.0460***					0.0460***				
		(7.51)					(7.51)				
Growth		-0.0326***					-0.0326***				
		(-7.77)					(-7.77)				
Board		0.0081					-0.0004				
		(1.43)					(-0.06)				
VOL		-0.0142***					-0.0155**				
		(-2.92)					(-2.53)				
Age		0.0555***					0.0575***				
		(8.78)					(7.34)				
Top1		-0.0036					-0.0106				
		(-0.54)					(-1.24)				
Shrcr		0.0201***					0.0267***				
		(3.00)					(3.07)				
Roa		0.0152***					0.0127*				
		(2.81)					(1.87)				
Dual		-0.0040					-0.0089				
		(-0.35)					(-0.61)				
Big4		-0.0233					-0.0069				
		(-0.98)					(-0.23)				
Audit		0.1089***					0.1116***				
		(4.77)					(3.70)				
Industry		Yes		Yes			Yes				
Year		Yes		Yes			Yes				
Constant		0.7243***		0.8431***			0.7821***				
		(7.74)		(5.49)			(6.89)				
Observations		20009		20009			20009				
R-sq		0.4460		0.5060			0.4543				
Adjusted R-sq		0.4442		0.4902			0.4513				
**Panel B**											
	(1)			(2)	(3)		(4)		(5)		
Variables	SYNCH_2			SYNCH_2	SYNCH_2		SYNCH		SYNCH		
EDT				-0.0289***	-0.0193**		-0.0229***		-0.0220***		
				(-3.12)	(-2.38)		(-2.83)		(-2.71)		
InsInvestor	-0.0292***				-0.0306***		-0.0272***		-0.0293***		
	(-4.90)				(-4.08)		(-3.62)		(-3.87)		
Lev	-0.1322***				-0.1336***		-0.1291***		-0.1289***		
	(-20.63)				(-15.73)		(-15.28)		(-14.90)		
Size	0.3432***				0.3280**		0.3388***		0.3376***		
	36.86				(27.63)		(28.83)		(28.14)		
Book_to_Market	0.0490***				0.0331***		0.0307***		0.0307***		
	(8.03)				(4.19)		(3.90)		(3.86)		
Growth	-0.0292***				-0.0320***		-0.0356***		-0.0353***		
	(-6.94)				(-5.29)		(-5.83)		(5.68)		
Board	0.0069				-0.0017		0.0003		-0.0012		
	(1.23)				(-0.24)		(0.05)		(-0.16)		
VOL	-0.0147***				-0.0166***		0.0046		-0.0158**		
	(-3.02)				(-2.70)		(0.80)		(-2.57)		
Age	0.0153**				0.0187**		0.0609***		0.0564***		
	(2.44)				(2.40)		(7.92)		(7.22)		
Top1	-0.0044				-0.0097		-0.0104		-0.0099		
	(-0.68)				(-1.14)		(-1.22)		(-1.15)		
Shrcr	0.0202***				0.0245***		0.0267***		0.0268***		
	(3.06)				(2.81)		(3.07)		(3.07)		
Roa	0.0163***				0.0137**		0.0130*		0.0157**		
	(3.01)				(2.00)		(1.92)		(2.25)		
Dual	0.0019				-0.0034		-0.0092		-0.0095		
	(0.16)				(-0.23)		(-0.63)		(-0.65)		
Big4	-0.0221				-0.0085		-0.0056		-0.0117		
	(-0.94)				(-0.28)		(-0.19)		(-0.39)		
Audit	0.1050***				0.1079***		0.1108***		0.1112***		
	(4.60)				(3.57)		(3.68)		(3.36)		
Industry	Yes			Yes	Yes		Yes		Yes		
Year	Yes			Yes	Yes		Yes		Yes		
Constant	0.6190***			0.7400***	0.7351***		0.7811***		0.7834***		
	(6.82)			(5.09)	(6.58)		(6.88)		(6.83)		
Observations	35596			35596	20009		20009		20009		
R-sq	0.4406			0.4060	0.4524		0.4541		0.4537		
Adjusted R-sq	0.4388			0.4519	0.4494		0.4511		0.4507		
**Panel C**											
Model	(1)		(2)			(3)		(4)		(5)	(6)
Variables	SYNCH		SYNCH			SYNCH		SYNCH		EDT	SYNCH
L2.EDT	-0.0129***										
	(-5.90)										
L3.EDT			-0.0183***								
			(-7.74)								
L4.EDT						-0.0203***					
						(-7.66)					
IV										0.2190***	
										(5.51)	
EDT								-0.1407***			
								(-7.20)			
EDT = (IV)											-0.1407***
											(-7.20)
Residuals E								0.0051			
								(1.04)			
InsInvestor	-0.0311		0.0259**			0.0274***		-0.1291***		0.1008***	-0.1291***
	(-2.04)		(1.44)			(1.26)		(-15.28)		(13.08)	(-15.28)
Lev	-0.1277***		-0.1441***			-0.1396***		0.3388***		0.3263**	0.3388***
	(-6.99)		(-6.90)			(-5.67)		(28.83)		(23.10)	(28.83)
Size	0.0252***		0.0275***			0.0238**		0.0307***		0.0607***	0.0307***
	(5.97)		(5.62)			(4.11)		(3.90)		(6.34)	(3.90)
Book_to_Market	-0.0096		-0.0026			-0.0098***		-0.0356***		-0.0316***	-0.0356***
	(-0.99)		(-0.24)			(-0.78)		(-5.83)		(-9.24)	(-5.83)
Growth	-0.0070***		-0.0107***			-0.0131***		0.0003		0.0002	0.0003
	(-1.76)		(-2.45)			(-2.63)		(0.05)		(0.04)	(0.05)
Board	0.0246		0.0244			0.0290		0.0046		0.0051	0.0046
	(1.79)		(1.55)			(1.57)		(0.80)		(0.72)	(0.80)
VOL	-0.0010***		-0.0020***			-0.0023***		0.0609***		0.0711**	0.0609***
	(-2.46)		(-4.30)			(-4.20)		(7.92)		(8.22)	(7.92)
Age	-0.0888		-0.0888***			-0.1513***		-0.0104		0.1120	-0.0104
	(-15.08)		(-15.08)			(-15.79)		(-1.22)		(0.10)	(-1.22)
Top1	-0.0302		-0.0258			0.0040		-0.0109		-0.0101	-0.0109
	(-1.29)		(-0.96)			(0.13)		(-0.29)		(-0.28)	(-0.29)
Shrcr	0.0526		0.0444			0.0423		0.0121		0.0101	0.0121
	(2.48)		(1.85)			(1.51)		(1.62)		(1.33)	(1.62)
Roa	-0.0005		-0.0052			-0.0109		-0.0473		-0.0553	-0.0473
	(-0.02)		(-0.16)			(-0.29)		(-3.00)		(-3.01)	(-3.00)
Dual	0.0068		0.0068			0.0121		0.0014		0.0020	0.0014
	(1.23)		(1.23)			(1.62)		(0.11)		(0.30)	(0.11)
Big4	-0.0218***		-0.0218***			-0.0473		0.0526		0.0650	0.0526
	(-1.81)		(-1.81)			(-3.00)		(2.48)		(2.71)	(2.48)
Audit	0.0084		0.0047			0.0014		-0.0020***		-0.0002	-0.0020***
	(0.84)		(0.43)			(0.11)		(-4.30)		(-0.30)	(-4.30)
Industry	Yes		Yes			Yes		Yes		Yes	Yes
Year	Yes		Yes			Yes		Yes		Yes	Yes
Constant	0.3186***		0.3594***			0.4290***		0.3308***		-0.6238***	0.3308***
	(0.51)		(0.91)			(2.04)		(3.09)		(-11.93)	(3.09)
Observations	20009		20009			20009		20009		20009	20009
R-sq	0.0448		0.0630			0.0784		0.0908		0.1138	0.0908
Adjusted R-sq	0.0388		0.0520			0.0618		0.0599		0.1146	0.0599

Note: All variables are as defined in Table 2

^*^p < 0.1

^**^p < 0.05

^***^p < 0.01.

### 5.5 Test of hypothesis

Panel A of Table 5 reports the core test results of the relationship between EDT and stock price synchronisation. In benchmark regression, this study adopts a progressive regression strategy. Column (1) controls the fixed effect of time and industry and tests the significance of the control variable when the dependent variable is the synchronisation of stock prices; column (2) only controls the fixed effect of time and industry, and the regression coefficient of EDT is -0.0312 and passes the 1% statistical significance test; in column (3), the control variable is included on the basis of the dependent variable, and the relevant regression coefficient is reduced (-0.0319), which may be due to the absorption of some factors affecting the synchronisation of stock prices after the control variable is included, but the significance remains unchanged (p less than 0.01). This means that the higher the degree of digital transformation of enterprises, the greater the reduction in the liquidity of enterprise stocks, and there is a significant negative correlation between the two. Therefore, the hypothesis H1a of this study is supported by empirical evidence. Overall, the results presented in Panel A of Table 5 provide strong support for H1a, suggesting that EDT has a significant negative correlation with stock price synchronisation.

We added EU as a regulatory variable to verify H2. As shown in Panel A of Table 6, column (1) controls for the fixed effects of time and industry and tests the significance when the independent variable is environmental uncertainty, showing a significance of 5%. Column (2) controls for the fixed effects of time and industry. The regression coefficient of the multiplier term (EDT_eu) of EDT and EU is -0.0450 and has passed the 1% statistical significance test, which shows that the greater the environmental uncertainty, the more enterprises with a high digital transformation process can reduce the synchronisation of stock prices and verifies the research hypothesis H2.

**Table 6:** Test of H2.

**Panel A**						
Model	(1)			(2)		
Variables	SYNCH			SYNCH		
EDT				-0.0062***		
				(-0.56)		
EU	-0.0738**			-0.0251**		
	(-3.65)			(2.22)		
EDT_eu				-0.0350**		
				(-2.55)		
InsInvestor	-0.0373***			-0.0372***		
	(-4.49)			(-4.49)		
Lev	-0.1234***			-0.1231***		
	(-13.82)			(-13.81)		
Size	0.3266***			0.3260**		
	(25.82)			(25.82)		
Book_to_Market	0.0356***			0.0358***		
	(4.19)			(4.21)		
Growth	-0.0371***			-0.0367***		
	(-5.89)			(-5.83)		
Board	0.0029			0.0031		
	(0.39)			(0.41)		
VOL	-0.0206***			-0.0208***		
	(-3.00)			(-3.03)		
Age	0.0462***			0.0460***		
	(5.09)			(5.07)		
Top1	-0.0115			-0.0113		
	(-1.25)			(-1.23)		
Shrcr	0.0170*			0.0173*		
	(1.84)			(1.87)		
Roa	0.0125*			0.0128*		
	(1.76)			(1.81)		
Dual	-0.0083			-0.0076		
	(-0.53)			(-0.49)		
Big4	-0.0339			-0.0326		
	(-0.96)			(-0.92)		
Audit	0.1195***			0.1189***		
	(3.83)			(3.82)		
Industry	Yes			Yes		
Year	Yes			Yes		
Constant	0.7718			0.7351		
	(6.31)			(6.58)		
Observations	20009			20009		
R-sq	0.4361			0.4365		
Adjusted R-sq	0.4325			0.4328		
**Panel B**						
Model	(1)			(2)		
Variables	SYNCH_2			SYNCH_2		
EDT				-0.0027*		
				(-0.24)		
EU	-0.0578*			-0.0252**		
	(-1.58)			(2.21) 0		
EDT_eu				-0.0343**		
				(-2.50)		
InsInvestor	-0.1211***			-0.0373***		
	(-3.03)			(-4.53)		
Lev	-0.2804***			-0.1270***		
	(-6.77)			(-14.21)		
Size_2	0.1121***			0.3174***		
	(12.57)			(24.95)		
Book_to_Market	0.0410			0.0398***		
	(1.57)			(4.66)		
Growth	-0.0455***			-0.0335***		
	(-4.46)			(-5.34)		
Board	0.0744**			0.0020		
	(2.00)			(0.27)		
VOL_2	-0.0084***			-0.0215***		
	(-7.27)			(-3.13)		
Age	-0.4787			0.0077		
	(-40.84)			(0.85)		
Top1	-0.0115			-0.0128		
	(-0.41)			(-1.39)		
Shrcr	0.0170**			0.0177*		
	(2.43)			(1.91)		
Roe	0.0125			0.0147**		
	(1.20)			(2.05)		
Dual	0.0457			-0.0044		
	(2.96)			(-0.28)		
Big4	0.0252			-0.0335		
	(0.73)			(-0.94)		
Audit	0.0584**			0.1159***		
	(2.21)			(3.71)		
Industry	Yes			Yes		
Year	Yes			Yes		
Constant	-0.5295***			0.6996		
	(-6.13)			(5.79)		
Observations	20009			20009		
R-sq	0.4311			0.4332		
Adjusted R-sq	0.4205			0.4296		
**Panel C**						
Model	(1)	(2)	(3)	(4)	(5)	(6)
Variables	SYNCH	SYNCH	SYNCH	SYNCH	EDT	SYNCH
L2.EDT	-0.0166*					
	(-1.40)					
L3.EDT		-0.0693***				
		(-7.74)				
L4.EDT			-0.0781***			
			(-8.01)			
IV					0.1190***	
					(6.01)	
EDT				-0.1577***		
				(-8.44)		
EDT = (IV)						-0.1577***
						(-8.44)
Residuals E				0.0053		
				(1.45)		
EU	-0.0237	-0.0856*	-0.0451	-0.0309	0.0050	-0.0309
	(-0.21)	(-0.83)	(-0.40)	(-0.72)	(0.02)	(-0.72)
EDT_eu	-0.0137*	-0.0157*	-0.0242*	-0.0290**	0.3263	-0.0290**
	(-0.32)	(-0.41)	(-0.59)	(10.83)	(0.10)	(10.83)
InsInvestor	0.1506	0.1618**	0.1939***	0.4407**	0.0557**	0.4407**
	(2.83)	(2.44)	(2.89)	(5.90)	(7.15)	(5.90)
Lev	-0.4563***	-0.4563***	-0.6798***	-0.0556***	-0.0303***	-0.0556***
	(-4.68)	(-10.06)	(-9.16)	(-6.33)	(-10.22)	(-6.33)
Size	0.2593***	0.2593***	0.2572**	0.0041	0.0004	0.0041
	(10.79)	(21.06)	(20.08)	(0.06)	(0.03)	(0.06)
Book_to_Market	-0.0872	-0.0006	-0.0471***	0.0066	0.0051	0.0066
	(-1.69)	(-0.24)	(-0.91)	(0.80)	(0.72)	(0.80)
Growth	-0.0364***	-0.0687***	-0.0893***	0.0707***	0.0710**	0.0707***
	(-1.76)	(-3.27)	(-3.77)	(7.01)	(8.02)	(7.01)
Board	0.1160	0.2036	0.2339	-0.0110	0.1220	-0.0110
	(1.79)	(1.55)	(3.51)	(-1.02)	(0.07)	(-1.02)
VOL	-0.0051***	-0.0142***	-0.0146***	-0.0409	-0.0103	-0.0409
	(-2.38)	(-4.30)	(-5.74)	(-0.33)	(-0.29)	(-0.33)
Age	-0.8060	-0.2577***	-0.3343***	0.0302	0.0109	0.0302
	(-20.71)	(-10.86)	(-11.32)	(1.12)	(1.13)	(1.12)
Top1	-0.2487	0.0722	0.0855	-0.0410	-0.0653	-0.0410
	(-1.94)	(0.70)	(0.74)	(-2.30)	(-4.01)	(-2.30)
Shrcr	0.3077***	0.3077	-0.2353	0.0015	0.0051	0.0015
	(2.73)	(2.70)	(-2.15)	(0.20)	(0.43)	(0.20)
Roa	-0.0655	-0.0876	-0.0350	0.0520	0.0651	0.0520
	(-0.43)	(-0.59)	(-0.21)	(2.49)	(2.31)	(2.49)
Dual	-0.0342	-0.0386	-0.0211	-0.0010	-0.0022	-0.0010
	(1.53)	(-1.50)	(-0.73)	(-0.30)	(-0.30)	(-0.30)
Big4	-0.0218***	-0.0492***	-0.0675	0.5308***	-1.6310***	0.5308***
	(-1.20)	(-0.88)	(-1.08)	(4.09)	(-12.73)	(4.09)
Audit	0.0332	0.0647	0.1110	0.6208***	-1.7110*	0.6208***
	(1.65)	(1.34)	(1.22)	(7.89)	(-4.93)	(7.89)
Industry	Yes	Yes	Yes	Yes	Yes	Yes
Year	Yes	Yes	Yes	Yes	Yes	Yes
Constant	-0.5393***	-0.9022***	-0.8290***	-0.2393***	0.5022***	-0.2393***
	(-0.53)	(-0.91)	(-1.22)	(-4.53)	(4.91)	(-4.53)
Observations	20009	20009	20009	20009	20009	20009
R-sq	0.1248	0.1384	0.1441	0.1602	0.1688	0.1602
Adjusted R-sq	0.1188	0.1210	0.1318	0.1554	0.1515	0.1554

Note: All variables are as defined in Table 2

^*^p < 0.1

^**^p < 0.05

^***^p < 0.01.

### 5.6 Robustness test

Panel B in Table 5 demonstrates the results of the robustness test, first replacing the measurement method of the explained variable (sync) from the original weighted average method of circulation market value with the weighted average method of total market value. After replacement, column (1) controls the fixed effect of time and industry and tests the significance of the control variable when the dependent variable is stock price synchronisation; column (2) only controls the fixed effect of time and industry, and the regression coefficient of EDT is -0.0179 and passes the statistical significance test of 1%; in column (3), the control variable set was included on the basis of dependent variables, the correlation regression coefficient was -0.0185, maintaining a significance of 1%, and the research hypothesis was still verified. Simultaneously, the key control variables were replaced, and the results after replacing the key control variables are listed in (4) and (5) of Panel B in Table 5. The results showed that the explained variables maintained a negative significance of 1%, thus supporting the research hypothesis.

However, the robustness test of the regulatory effect model still replaces the measurement method of the explained variable stock price synchronisation (sync). The original weighted average method of the circulation market value was changed to the weighted average method of the total market value. After replacement, as shown in Panel B of Table 6, column (1) controls the fixed effect of time and industry and tests the significance when the independent variable is environmental uncertainty and the dependent variable is stock price synchronisation. Column (2) controls the fixed effects of time and industry and adds the EDT index and the intersection and multiplication term (edt_eu) of EDT and environmental uncertainty. After testing, the regression coefficient of the intersection term was -0.0293 and passed the 5% statistical significance test. Thus, the research hypothesis was verified. However, as shown in Panel C of Table 6, using the explanatory variable (EDT) with a lag of 2–4 periods, it was found that the cross-multiplication term EDT_EU maintains a significance of 10% and the regression coefficient is negative, which shows that environmental uncertainty plays a negative regulatory role in the action mechanism of digital transformation on stock price synchronisation, and thus hypothesis H2 is verified.

### 5.7 Endogenous test

1. **Lagged explanatory variables**
  Does digital transformation inhibit stock price synchronisation, or does stock price synchronisation give birth to the promotion of digital transformation? There may be endogenous problems caused by reverse causality between digital transformation and stock price synchronisation. Considering that the digital transformation of enterprises is a comprehensive application of digital technology, when enterprises conduct the strategic deployment of digital transformation or make relevant decisions (such as investment in related manufacturing equipment or software and system applications), it takes some time for its driving effect to have a relationship. This study uses lag explanatory and instrumental variables to deal with endogenous problems. As shown in Panel C of Tables 5 and 6, because digital transformation requires a certain time response and adaptation, the results still maintain 1% significance by testing the lag of 2–4 explanatory variables (EDT).
2. Instrumental variables
  The instrumental variable method is used to further reduce endogenous interference and enhance the robustness of core research conclusions. In terms of the selection of tool variables, the works of Zhao Chenyu et al. (2021) and Yi Luxia et al. (2021) were referred, and the proportion of the word frequency of 20 digital application technology keywords in the manufacturing industry to the total word frequency was used as a tool variable for the endogenous test [44, 45].
  To determine the endogeneity of the digital transformation variables, the digital transformation (EDT) was regressed on all exogenous variables (IV, Size, Board, Roa, Book_to_Market, VOL, InsInvestor, Age, Growth, Lev, Shrcr, Top1, Big4, Dual, Audit) to obtain the residual E. In the second step, the residual E was added as an explanatory variable in model (6). The regression coefficient ρof E was tested to check if it is 0. The result showed that the regression coefficient ρ is 0.0104, thus the original hypothesis is rejected, indicating an endogeneity problem. The correlation between the selected instrumental variables and the endogenous explanatory variables was examined.
  Next, we regressed endogenous variables (EDT) on all exogenous variables, including instrumental variables. The results of the test found that the regression coefficient of IV was significant at the 1% level, indicating that the instrumental variable (IV) is correlated with the endogenous variable (EDT) and is able to explain part of the message of digital transformation.
  Furthermore, we regressed all exogenous variables using the estimates of EDT as proxies. Panel C of Tables 5 and 6 shows the results of the endogeneity test for the instrumental variables. It can be observed that the robustness of the model is still guaranteed after the inclusion of instrumental variables.
  Meanwhile, the Sargan test with p-values of 0.0534 and 0.0520 (>0.05) did not reject the original hypothesis of valid overidentification, which suggests that the instrumental variables are valid. Therefore, the instrumental variables approach is able to reduce endogenous variables.
3. Heckman two-stage model
  The Heckman two-stage model is used to test the endogeneity problem. To reduce endogeneity interference, we first use a binary choice model to calculate the inverse Mills ratio (IMR). The setup is as follows:
$$\mathrm{Pr}(\mathrm{EDT}=1|X_{n})=d_{0}+d_{1}{\mathrm{Size}}_{it}+d_{2}{\mathrm{Age}}_{\mathrm{it}}+d_{3}{\mathrm{BM}}_{\mathrm{it}}+d_{4}{\mathrm{Growth}}_{\mathrm{it}}+d_{5}{\mathrm{Lev}}_{\mathrm{it}}+d_{6}{\mathrm{Shrcr}}_{\mathrm{it}}+d_{7}{\mathrm{VOL}}_{\mathrm{it}}+d_{8}{\mathrm{ROA}}_{\mathrm{it}}+d_{9}{\mathrm{Inshold}}_{it}+d_{10}{\mathrm{Board}}_{it}+d_{11}{\mathrm{Dual}}_{it}+d_{12}{\mathrm{Big4}}_{it}+\varepsilon_{it}$$ (8)

In this case, the digital transformation is divided into two groups according to the mean value, and the group with the larger value is 1, otherwise it is 0.

Then, the inverse Mills ratio was put into models (5), (6), and (7). The coefficient of EDT in column (7) of group C in Tables 5 and 6 is significant, as is the coefficient of IMR, indicating the presence of endogeneity caused by sample self-selection. However, the sign of the coefficient of EDT is the same and significant as in the previous study, which indicates that after controlling for the problem of self-selection, the previous findings are still valid, the conclusions are still relatively robust, and the research hypotheses H1A and H2A are tested.

## 6 Influence mechanism analysis

The content of this chapter focuses on the factors through which the implementation of digital transformation by firms in turn improves the information efficiency of the capital market and reduces share price synchronisation. In order to accomplish this goal, it is necessary to test the influence of several factors on the main model with the help of mediating effects, several factors related to digital transformation will be selected as mediating variables, and their significance in the model of digital transformation and capital market information efficiency will be tested to determine whether there are these factors affecting the information efficiency of the capital market through the digital transformation of enterprises.

The main reasons for the synchronisation of stock prices were low analyst coverage and investor sentiment. As important stakeholders of listed enterprises, analyst coverage shares an important relationship with the enterprise’s business activities, financial situation, and information environment, consequently affecting the enterprise’s stock market performance. With this understanding, this study examines the factors that influence stock price synchronisation and utilizes the intermediary effect to assess the internal effect of digital transformation on stock price synchronisation.

### 6.1 Analyst coverage

As an essential information intermediary connecting listed enterprises with external investors, analysts play a significant role in reducing information asymmetry and improving capital market efficiency, and the content and quality of enterprise information are important factors affecting their attention. Wu Fei et al. (2021) found that enterprises with higher analyst attention can enhance the volatility of their stock, which is driven by digital transformation [46]. The increase in stock price volatility of enterprises that have implemented digital transformation also has an inhibitory effect on the synchronisation of stock prices in the capital market, which makes it significantly different from other enterprises, thereby improving the information efficiency of the capital market. Martens et al. (2021) proposed that financial analysts not only reduce information asymmetry between enterprises and capital market participants but also promote the generation of business intelligence through feedback and information spillover between enterprises [47]. Therefore, the test path was ‘digital transformation→analyst coverage→stock price synchronisation’.

Panel A of Table 7 shows the results of the test of the mechanism of analyst focus. First, we evaluated the relationship between digital transformation and the information efficiency of the capital market (EDT→synch). The regression coefficient of digital transformation (EDT) was -0.0325 and the T value was -2.89, which was significant at the 1% level, indicating that digital transformation significantly reduced the synchronisation of stock prices. Second, we assessed the relationship between digital transformation and analyst coverage (EDT→follow). The regression coefficient of digital transformation (EDT) was 0.0866 and the T-value was 6.51, indicating that digital transformation had increased analyst coverage, which was significant at the 5% level. Finally, the relationship between digital transformation, analyst coverage, and stock price synchronisation was evaluated. The regression coefficient of digital transformation (EDT) was -0.0337, and the T value was -2.22. The regression coefficient of analyst coverage was 0.0155, and the T value was 1.92, which was significant at the 10% level. According to the principle of the intermediary effect test, analysts pay attention to the intermediary effect between digital transformation and stock price synchronisation. It can be seen that the mechanism of the digital transformation of companies on information efficiency is analyst attention, which means that the digital transformation of companies increases the attention of analysts to the company, and companies with high analyst attention generate lower share price synchronisation, which means that the share price is more reflective of the company’s own specific changes and information, and these result in the ultimate improvement of the information efficiency of the capital market, in which analyst attention plays a mediating role.

**Table 7:** Analysis of influence mechanism.

**Panel A**						
Model	(1)		(2)		(3)	
Variables	SYNCH		Follow		SYNCH	
EDT	-0.0225***		0.0766**		-0.0206**	
	(-2.78)		(6.21)		(-2.22)	
Follow					0.0155*	
					(1.92)	
InsInvestor	-0.0300***		0.1949***		-0.0259***	
	(-3.98)		(7.78)		(-2.95)	
Lev	-0.1285***		-0.0189		-0.1204***	
	(-15.19)		(-1.20)		(-11.69)	
Size	0.3373***		0.2360***		0.3209***	
	(28.60)		(11.43)		(23.24)	
Book_to_Market	0.0303***		-0.1418***		0.0367***	
	(3.85)		(-11.09)		(3.91)	
Growth	-0.0353***		0.0226**		-0.0449***	
	(-5.80)		(2.29)		(-5.89)	
Board	-0.0004		0.0814***		-0.0058	
	(-0.06)		(6.36)		(-0.71)	
VOL	-0.0155**		-0.0795***		-0.0146**	
	(-2.53)		(-8.26)		(-2.02)	
Age	0.0575***		-0.0832***		0.0681***	
	(7.34)		(-5.71)		(7.22)	
Top1	-0.0106		-0.0321**		-0.0009	
	(-1.24)		(-2.24)		(-0.09)	
Shrcr	0.0267***		0.0209		0.0239**	
	(3.07)		(1.48)		(2.36)	
Roa	0.0127*		0.3285***		0.0135	
	(1.87)		(24.10)		(1.35)	
Dual	-0.0089		0.0825***		-0.0223	
	(-0.61)		(3.24)		(-1.29)	
Big4	-0.0069***		0.4584***		-0.0294	
	(-0.23)		(9.42)		(-0.94)	
Audit	0.1116***		0.0148		0.0452	
	(3.70)		(0.22)		(0.93)	
Industry	Yes		Yes		Yes	
Year	Yes		Yes		Yes	
Constant	-0.0931***		0.0097***		0.7864***	
	(-7.95)		(6.05)		(5.76)	
Observations	20009		20009		20009	
R-sq	0.4543		0.2378		0.4499	
Adjusted R-sq	0.4513		0.2320		0.4457	
**Panel B**						
Model		(1)		(2)		(3)
Variables		SYNCH		Innov		SYNCH
EDT		-0.0225***		0.0329**		-0.0267***
		(-2.78)		(2.02)		(-3.12)
Innov						-0.0070
						(-0.95)
InsInvestor		-0.0300***		-0.0562***		-0.0373***
		(-3.98)		(-3.51)		(-4.43)
Lev		-0.1285***		-0.0740***		-0.1193***
		(-15.19)		(-4.08)		(-13.06)
Size		0.3373***		0.0344		0.3190***
		(28.60)		(1.56)		(24.15)
Book_to_Market		0.0303***		0.0006***		0.0415***
		(3.85)		(0.05)		(4.67)
Growth		-0.0353***		0.0273***		-0.0416***
		(-5.80)		(3.21)		(-6.55)
Board		-0.0004		-0.0015		0.0018
		(-0.06)		(-0.10)		(0.23)
VOL		-0.0155**		-0.0155		-0.0201***
		(-2.53)		(-1.48)		(-2.89)
Age		0.0575***		-0.0685***		0.0453***
		(7.34)		(-3.66)		(4.86)
Top1		-0.0106		-0.0272**		-0.0069
		(-1.24)		(-1.53)		(-0.74)
Shrcr		0.0267***		0.0194		0.0159*
		(3.07)		(1.13)		(1.67)
Roa		0.0127*		-0.0184***		0.0158**
		(1.87)		(-1.64)		(2.20)
Dual		-0.0089		-0.0136***		-0.0055
		(-0.61)		(-0.46)		(-0.35)
Big4		-0.0069		-0.0808***		-0.0274
		(-0.23)		(-1.21)		(-0.75)
Audit		0.1116***		0.0172		0.1128***
		(3.70)		(0.33)		(3.57)
Industry		Yes		Yes		Yes
Year		Yes		Yes		Yes
Constant		-0.0931***		0.0649		0.7828***
		(-7.95)		(0.25)		(6.28)
Observations		20009		16664		16664
R-sq		0.4543		0.0458		0.4351
Adjusted R-sq		0.4513		0.0395		0.4313

Note: All variables are as defined in Table 2

^*^p < 0.1

^**^p < 0.05

^***^p < 0.01.

### 6.2 Investment in technological innovation

The digital transformation of enterprises reduces the risk involved in technological innovation, improves the performance of technological innovation, and enhances the willingness of enterprises to innovate. An increase in enterprise innovation investment will increase the stock price of enterprises and restrain the synchronisation of stock prices. Therefore, the test path was‘digital transformation →technological innovation→stock price synchronisation’.

Panel B of Table 7 shows the results of the test of the mechanism of technological innovation. First, we assessed the relationship between digital transformation and stock price synchronisation (EDT→synch). The regression coefficient of digital transformation (EDT) was -0.0308 and the T value was -2.98, which was significant at the 1% level, indicating that digital transformation significantly reduced stock price synchronisation. Second, the relationship between digital transformation and innovation investment (EDT→innov) was evaluated. The regression coefficient of digital transformation (EDT) was 0.0329 and the T-value was 2.02, which was significant at the 5% level, indicating that digital transformation significantly increased innovation investment. Finally, we assessed the relationship between digital transformation, innovation investment, and stock price synchronisation. The regression coefficient of digital transformation (EDT) was -0.0407, and the T value was -3.62. The regression coefficient of innovation investment (innov) was -0.0070, and the T value was -0.95. These results indicate that there is no significant intermediary effect between digital transformation and stock price synchronisation. A plausible reason why technological innovation investment does not reduce the synchronisation of stock prices may be that technological innovation investment does not necessarily bring greater volatility and improvement to the stock prices of enterprises, which does not necessarily result in significant stock price heterogeneity; therefore, it does not reflect a significant intermediary effect.

## 7 Additional analyses

### 7.1 Enterprise size

The research sample was divided into large-scale enterprises and small- and medium-sized enterprises according to the size of the enterprise, and Panel A of Table 8 shows the regression result of distinguishing the size of enterprises. It suggests that the digital transformation of small and medium-sized enterprises has a better relationship with the synchronisation of stock prices. Digital technology breaks the temporal and spatial constraints of resource allocation and drives the sharing economy. Small and medium-sized enterprises conduct production and manufacturing, product testing, and logistics distribution through the sharing economy platform, and scientific research institutes conduct joint innovation to significantly improve the return on asset investment and innovation efficiency with lower investment, reduce the dependence on financing channel funds, and then effectively enhance the enterprise’s stock price and its heterogeneity and restrain the synchronisation of stock prices.

**Table 8:** Additional analysis.

**Panel A**									
Model		Sample group of large scale enterprise			Sample group of small and medium-sized enterprises				
Variables		(1) SYNCH		(2) SYNCH	(3) SYNCH		(4) SYNCH		
EDT		-0.0225		-0.0062	-0.1027***		-0.0765***		
		(-1.78)		(-0.56)	(-10.67)		(-6.12**		
EU				-0.0251**			-0.0283**		
				(2.22)			(-2.19)		
EDT_eu				-0.0350			-0.0457***		
				(-1.55)			(-2.96)		
InsInvestor		-0.0300***		-0.0372***	-0.0093***		-0.0099***		
		(-3.98)		(-4.49)	(-0.99)		(-1.05)		
Lev		-0.1285***		-0.1231***	-0.1067***		-0.1060***		
		(-15.19)		(-13.81)	(-10.32)		(-10.17)		
Size		0.3373***		0.3260***	0.2865***		0.2869***		
		(28.60)		(25.82)	(21.97)		(21.67)		
Book_to_Market		0.0303***		0.0358***	0.0045		0.0062		
		(3.85)		(4.21)	(0.50)		(0.68)		
Growth		-0.0353***		-0.0367***	-0.0411***		-0.0409***		
		(-5.80)		(-5.83)	(-5.53)		(-5.48)		
Board		-0.0004		0.0031	0.0334		0.0337		
		(-0.06)		(0.41)	(3.97)		(3.93)		
VOL		-0.0155**		-0.0208***	-0.0745***		-0.0753***		
		(-2.53)		(-3.03)	(-9.07)		(-9.10)		
Age		0.0575***		0.0460***	-0.0487***		-0.0497***		
		(7.34)		(5.07)	(-4.06)		(-4.05)		
Top1		-0.0106		-0.0113	-0.0016		-0.0016		
		(-1.24)		(-1.23)	(-0.14)		(-0.14)		
Shrcr		0.0267***		0.0173*	-0.0212*		-0.0212*		
		(3.07)		(1.87)	(-1.85)		(-1.85)		
Roa		0.0127*		0.0127*	0.0085		0.0093		
		(1.87)		(1.87)	(1.05)		(1.14)		
Dual		-0.0089		-0.0089	-0.0360**		-0.0356**		
		(-0.0061)		(-0.61)	(-2.00)		(-1.97)		
Big4		-0.0069		-0.0069	-0.0426		-0.0430		
		(-0.23)		(-0.23)	(-1.11)		(-1.10)		
Audit		0.1116***		0.1116***	0.1751***		0.1732***		
		(3.70)		(3.70)	(4.85)		(4.77)		
Industry		Yes		Yes	Yes		Yes		
Year		Yes		Yes	Yes		Yes		
Constant		0.7821***		0.7748***	0.3447***		0.3509***		
		(6.89)		(6.34)	(2.62)		(2.67)		
Observations		20009		17526	17830		17526		
R-sq		0.4543		0.4365	0.1674		0.1654		
Adjusted R-sq		0.4513		0.4328	0.1622		0.1601		
**Panel B**							
Model	Sample group of state-owned enterprises				Sample group of non-state-owned enterprises		
Variables	(1)SYNCH	(2)SYNCH			(3)SYNCH		(4)SYNCH
EDT	-0.0116	-0.0096			-0.0406***		-0.0359***
	(-1.15)	(-0.68)			(-3.20)		(-3.35)
EU		-0.0336**					-0.0486***
		(-3.17)					(-3.04)
EDT_eu		-0.1309					-0.0629***
		(-0.83)					(-3.05)
InsInvestor	-0.0243***	-0.0336**			-0.0413***		-0.0445***
	(-2.54)	(-3.17)			(-3.47)		(-3.46)
Lev	-0.1343***	-0.1309**			-0.1110***		-0.1060***
	(-12.90)	(-11.83)			(-8.34)		(-7.70)
Size	0.3328***	0.3136***			0.3232***		0.3214***
	(21.78)	(18.94)			(19.73)		(18.57)
Book_to_Market	0.0326***	0.0394***			0.0324		0.0338***
	(3.23)	(3.51)			(2.75)		(2.75)
Growth	-0.0323***	-0.0350***			-0.0409***		-0.0392***
	(-4.38)	(-4.52)			(-4.05)		(-3.83)
Board	0.0008***	0.0013			-0.0009		0.0063
	(0.09)	(0.14)			(-0.09)		(0.53)
VOL	0.0006**	-0.0016***			-0.0461***		-0.0541***
	(0.08)	(-0.18)			(-4.50)		(-4.84)
Age	0.0700***	0.0625***			0.0374***		0.0245*
	(6.87)	(5.10)			(2.99)		(1.72)
Top1	-0.0091	-0.0095			-0.0132		-0.0205
	(-0.86)	(-0.82)			(-0.95)		(-1.39)
Shrcr	0.0248***	0.0143*			0.0227*		0.0205
	(2.32)	(1.26)			(1.54)		(1.31)
Roa	0.0161*	0.0147*			0.0039		0.0059
	(1.98)	(1.72)			(0.33)		(0.48)
Dual	0.0050	0.0020			-0.0439**		-0.0361
	(0.29)	(0.11)			(-1.71)		(-1.35)
Big4	-0.0290	-0.0697			0.0037		-0.0035
	(-0.69)	(-1.34)			(0.09)		(-0.08)
Audit	0.1298***	0.1469***			0.0759***		0.0773
	(3.51)	(3.80)			(1.52)		(1.53)
Industry	Yes	Yes			Yes		Yes
Year	Yes	Yes			Yes		Yes
Constant	0.4894***	0.4550***			0.9271***		0.9255***
	(2.79)	(2.36)			(6.46)		(6.05)
Observations	12443	10635			7566		6886
R-sq	0.4504	0.4293			0.4729		0.4604
Adjusted R-sq	0.4455	0.4232			0.4653		0.4517

Note: All variables are as defined in Table 2

^*^p < 0.1

^**^p < 0.05

^***^p < 0.01.

### 7.2 Nature of property rights

Given the particular nature of the property rights of enterprises in China, there may be differences in the association between digital transformation and the synchronisation of stock prices among enterprises with different property rights. When the state has ownership or control over enterprises, they are classified as state-owned enterprises; otherwise, they are classified as non-state-owned enterprises. Panel B in Table 8 shows the results of the analysis of the nature of different enterprise property rights. It can be seen that the inhibitory effect of digital transformation on stock price synchronisation is substantially different between state-owned enterprises and non-state-owned enterprises, compared with state-owned enterprises, the inhibitory effect of digital transformation on stock price synchronisation is more effective in non-state-owned enterprises. In sharp contrast, non-state-owned enterprises have serious resource constraints, respond quickly to market changes, actively seize the historical opportunities of the digital economy to promote the high-quality development of enterprises, and thoroughly implement a digital transformation strategy so that the digital transformation of non-state-owned enterprises can have a more positive effect on restraining the synchronisation of stock prices.

## 8 Conclusion

Based on data on A-share-listed enterprises in Shenzhen and Shanghai from 2007 to 2022, this study empirically examined the association between digital transformation and stock price synchronisation. It was found that the digital transformation of enterprises significantly reduces the synchronisation of stock prices and improves the information efficiency of the capital market. Environmental uncertainty plays a moderating role in this relationship. In terms of the heterogeneous characteristics of enterprise size and property rights, it was found that the inhibitory effect of digital transformation on stock price synchronisation is better for non-state-owned enterprises and small and medium-sized enterprises.

Through this study, we can give some reference to the digital transformation work of enterprise managers and also have more reference to the strategic deployment and formulation of digital transformation work. For different enterprises of different nature and size, we need to put forward targeted transformation suggestions and plans that will help enterprises better realise digital transformation and better reflect the advantages and importance of digital transformation.

### 8.1 Policy and managerial implications

Based on the above conclusions, this study has the following policy suggestions: First, digital transformation should be supported. This study finds that digital transformation has a positive inhibitory effect on stock price synchronisation. Second, the effective path of the transmission mechanism of digital transformation should be broadened, and the ‘blocking point’ should be opened. Enterprises need to deepen the degree of digital transformation. They also need to increase innovation investment to enhance innovation efficiency. Third, a targeted policy on digital transformation should be introduced. To seize the historical opportunity for the development of digital technology, it is necessary for government departments to formulate differentiated policies and vigorously promote the digital transformation of non-state-owned enterprises and small- and medium-sized enterprises.

### 8.2 Study limitations

This study has limitations despite its theoretical contribution. These limitations have to be removed by some appropriate additions. This paper has investigated the impact of digital transformation on the capital market for enterprises of different natures and sizes, but it has not done more research on the different performance of enterprises with different business conditions or types in the relationship between digital transformation and the capital market. At the same time, the study of the relationship between digital transformation and capital market of enterprises should not only focus on information efficiency. In addition, there may be some limitations in the data definitions of the variables due to the difficulty in obtaining data.

### 8.3 Future prospects

Although this study has been completed, there are many parts that deserve to be studied in depth. In future research work, we will continue to explore the impact of digital transformation on other aspects of the capital market and to compare the different ways in which different types and natures of firms perform differently in the impact of digital transformation on the capital market. It will also focus on the factors within firms that affect the performance of digital transformation efforts and further explore digital transformation at the micro level. In addition, other methods of defining digital transformation variables will be explored and more data will be collected to aid the research.

## Supporting information

S1 Table List of keywords for enterprise digital transformation. (XLSX)
