# Correction: Assessing the impact of digital transformation on capital market information efficiency under environmental uncertainty: Evidence from China

**DOI:** 10.1371/journal.pone.0325842

**Published:** 2025-05-29

**Authors:** Tao Feng, Xiaohuan Dong, Yueyun Wang

The Funding statement for this paper is incorrect. The correct Funding statement is as follows: First-class undergraduate course construction project of Xinhua College of Ningxia University (No. 2021yjyl016) awarded to Y.W.; Funded by Scientific research project of Ningxia Education Department (No. NYG2024228) awarded to Y.W; and the Scientific research project of Ningxia Education Department. (No. NYG2024228) awarded to Y.W.
